# Army Recollections

**Published:** 1888-08-04

**Authors:** 


### Army Recollections.

### I.—MILITARY PUNISHMENTS, ANCIENT AND MODERN.

Though this looks a somewhat ambitious heading, I have no intention of writing up to, or, much less, exhausting it. I have neither the power nor the means of doing so. I will, therefore, merely relate here my own experiences in this line, and then leave to others the descriptions of them I am unable to supply. It is conceded on all hands that the condition of the soldier is an exceptional one, and that it must be met or regulated by exceptional legislation. He has ever been regarded as one who requires more judicious handling or greater restraint than his civilian brother, for his life is always a monotonous, and at times even a very irksome one—"a daily routine of drill, parade, guard-mounting, relieved by long hours of listless indolence, and vapid conversation at mess and elsewhere. Nobody who has looked behind the scenes can (says the late Rev. F. W. Roberston in "Blackwood's Magazine" for January, 1866) pretend to say that under existing circumstances military life is in this country any other than a life of the most pitiable idleness, from the deadening influence of which, if individuals succeed in escaping, they owe their deliverance to their own strength of will, and to the power which strong will gives of holding our course, in spite of the strongest public inducements to the contrary."

If that be so in England, how much more is it likely to be so in India, where the heat is often so potent or continuous as to be dangerous to life, and where the soldier has, for his own sake, to spend some nine months or so of every year boxed up like a bear between four white walls? He has in this cheering recess little else than his dog or his parrot to converse with—for his wearied associates have long since exhausted their stores of jest or fun—and then spend the remainder of his time between the bazaar and the canteen. No wonder that he sometimes gives way to drink, or becomes insubordinate. The wonder would be, indeed, did he not do so; but, all the same, he must be kept within bounds, and how to do this fairly, and at the same time justly, is the problem his masters have to face. How they contrive to solve it so well was often to me a source of surprise, but then their powers are enormous; they wield the whip with a vengeance, and we all know that "what's in the Captain but a choleric word, is in the soldier downright blasphemy."

The famous Roman military writer, Vegetius, said eighteen hundred years ago or more in his "De Re Militari," lib. 11, cap. 4, that "Laudabiliores tamen dures sunt quorum exercitus ad modestiam laboret cessio instituit quam ille quorum milites ad obentium. Supplicide formido compellit," we all know that example is better that precept. "Let the commander do his duty (says Sir Charles the Napier), that is the great secret. Neither rewards nor punishments have so much effect as example," and dwelling on the secret of Havelock's mastery over his men, Colonel Malleson truly observes, ("Essays and Lectures," vol. 1, p. 174), that "there is probably no class more quick-witted, more imbued with a sense of their own rights, more jealous of maintaining them than the private soldiers." That is so, and I cannot help thinking that if Thomas Atkins were shown a better example in high quarters than is now always the case, he would, bar the liquor, be a more manageable, or less exacting personage, than he now sometimes is.

As to my own personal experience, it is necessarily limited, for I entered the service many years after the grosser punishments of the old regime had been abolished, and fifty strokes of the cat on the bare back is the worst form of it that I have witnessed. This amount could, I think, hurt seriously no sound man, anyhow, and albeit I have witnessed many of those inflictions, I saw no hurt or damage from it; and this, too, was the opinion of all the medical officers I have conversed with on the subject. That grave, nay, irreparable injuries were, however, inflicted on soldiers by these big punishments in former days, cannot be questioned, and I have often heard that the horrible practice of keel-hauling—which was at one time legalised in our navy—has sent many prematurely to their graves. Of this, however, I know nothing, but instances wherein death resulted from protracted floggings are mentioned in "The Literary Life and Correspondence of the Countess of Blessington," as well as in the "Autobiography of James Silk Buckingham," vol. i., p. 158; and the Hounslow case or its literature will enable the curious reader to supply my deficiencies in this respect. Thanks to the late Mr. Wakley, this was so threshed out at the time as to exhaust the subject. It opened the eyes of the public to the needless and often grotesque cruelties to which their brethren were subjected, and led, as I believe, to that final closure of this question that has given so much satisfaction to the friends of humanity as well as to those of the soldier classes everywhere.

"To govern those whose mental and physical energies are required for our subsistence or support by the fear of the lash alone, is (says the late Sir W. Sleeman, the suppressor of Thuggee, in India—'The Rambles and Recollections of a Bengal Officer,' vol. ii., p. 434) so easy, so simple a mode of binding them to our will . . . that it is not at all surprising to find so many of those who have become accustomed to it, and are not themselves liable to have the lash inflicted upon them, advocating its free use. In China the Emperor has his generals flogged, and finds the lash so efficacious in binding them to his will that nothing would persuade him that it could be safely dispensed with! In some parts of Germany they had the officers flogged, and princes and generals found this so very efficacious in making those act in conformity to their will that they found it difficult to believe that any army could be well managed without it. In other Christian armies the officers are exempted from the lash, but they use it freely upon all under them, and it would be extremely difficult to convince the greater part of these officers that the free use of the lash is not indespensably necessary,—nay, that the men themselves do not like to be flogged; as eels like to be skinned when they once get used to it. Ask the slave holders of the Southern States of America (he continues) whether any society can be well constituted unless the greater part of those upon the sweat of whose brow the community depends for its subsistence are made by law liable to be bought, sold, and driven to their daily labour with the lash. They will one and all say, No; and yet there are, doubtless, many very excellent and amiable persons among these slave-holders. If our army, as at present constituted, cannot do without the free use of the lash, let its conditions be altered, for no nation with free institutions should suffer its soldiers to be flogged."

No one can wade through the Punic War of Procopius without being startled or disgusted at the frequency with which the cross was resorted to by the Carthagenians for the punishment of their soldiers. They were as wedded to it as we were at one time to the lash, but they administered it much more impartially than we did. They strung or nailed up their defaulting or unfortunate generals and admirals, as we did our Byngs or Sackvilles, *pour encourger les autres*; and they were even harder on spies than the Americans were on Major André. Hannibal is said to have crucified a guide who either consciously or unconsciously misled him; and as to the Romans and their military punishments, Gibbon tells us—"The Decline and Fall," vol. i., p. 5—that "the centurions were authorized to chastise with blows; the generals had right to punish with death; and it was an inflexible maxim of Roman discipline that a good soldier should dread his officers far more than the enemy."

So much for the ancient usage on this point: let us now look for its counterpart in modern times. Mr. Sutherland Edwards tells us, in his "The Russians at Home," p. 131, that the Russian officers kick and cuff their men; and I have somewhere seen it stated that though "in France there is no such thing as flogging, either in the prisons or in the government schools, or in the army; but the Algerian soldiers, though not flogged, used to be tortured to death (and perhaps are now) by being suspended by the arm from a tree, with the toe of one foot resting on a cannon ball, and exposure of the face and eyes to a scorching sun." Whether this is so or not I can't say, and the following extract from a master-hand the late Sir W. Napier, will appropriately bring this subject to a close. Adverting at some length to the treatment of our soldiers, and after paying a high compliment to the Irish as soldiers, he says ("Biography," vol. ii, p. 241) that "the Russians use the knout. The cane is used by the Austrians, and is not entirely banished from the Russian army. The Swedes, the Danes inflict blows. The Portuguese and Spaniards strike on the breast and back with the flat of a sword—a terrible punishment. The French hang heavy weights to the legs, and use a variety of punishments which our soldiers would consider more degrading than the lash; ' and we may all congratulate ourselves that such degrading practices are no longer possible in our midst.

*(To be continued.)*

